# Ultrasensitive and Real-Time Detection of Kanamycin Residues in Milk Using an Aptasensor Based on Microfluidic Capacitive Strategy

**DOI:** 10.3390/bios15050322

**Published:** 2025-05-18

**Authors:** Weidong Zheng, Jun Chai, Jayne Wu, Jian Zhang, Haochen Qi

**Affiliations:** 1 College of Electrical and Electronic Engineering, Wenzhou University, Wenzhou 325035, China; 2 Department of Electrical Engineering and Computer Science, The University of Tennessee, Knoxville, TN 37996, USA

**Keywords:** kanamycin, interface capacitance, microfluidic enrichment, aptasensor

## Abstract

Kanamycin (KanR) is a widely used antibiotic in human and veterinary medicine, as well as in food production and livestock breeding. However, its environmental residue and bioaccumulation in the food chain pose a great threat to human health. A real-time and sensitive aptasensor is developed for KanR detection based on a gold interdigitated electrode (IDE). A microfluidic alternating current electrothermal (ACET) effect is employed for rapid directional manipulation and enrichment of KanR molecules. As an ultrasensitive indicator, solid–liquid capacitance is adopted to reflect the tiny change on the IDE surface caused by target adsorption. The overall detection takes only 60 s from sample to result, and a wide linear detection range of 0.1 fM~1 pM, an ultra-low detection limit of 16.56 aM, and a high selectivity of 7752:1 are simultaneously achieved, with 5 times of repeated use and the shelf life of 10 days. Furthermore, the aptasensor shows excellent practicability in milk samples, with the spiked recovery rate ranging from 86.90% to 116.17%. This aptasensor with the detecting strategy provides a rapid, convenient, and cost-effective solution for real-time monitoring of KanR.

## 1. Introduction

Kanamycin (KanR, C_18_H_38_N_4_O_15_S) is a common aminoglycoside antibiotic derived from the fermentation broth of streptomyces kanamyceticus [1,2], which is widely utilized due to its low price, high solubility in water, and broad-spectrum antibacterial properties in the livestock industry for treating infections caused by various bacteria [3]. Initially, kanamycin was used solely for disease treatment; however, its applications have gradually expanded into animal feed as a growth promoter and prophylactic agent, with its consumption increasing significantly over the last decade [4]. Studies have revealed that the abuse of kanamycin gives rise to drug resistance among pathogenic strains. Moreover, approximately 30%~90% of the ingested kanamycin is excreted into the environment, leading to contamination due to its low absorptivity for animals [5]. Even worse, its residues in animal-derived foods can lead to bioaccumulation in the human body [6], potentially causing various toxic effects such as ototoxicity, nephrotoxicity, and hepatotoxicity [7,8]. Therefore, numerous countries, such as the European Union, the United States, Japan, South Korea, and China, have stated the upper limits of residue in animal-derived foods, typically from 100 to 600 μg/kg [9,10,11]. Hence, the development of rapid and sensitive detection techniques for KanR residues is essential for food and environmental safety, and can even facilitate clinical diagnosis.

Traditional methods for KanR detection include enzyme-linked immunosorbent assay [12,13], high-performance liquid chromatography [14], liquid chromatography–mass spectrometry [15], capillary electrophoresis [16], surface plasmon resonance [17], and microbial detection methods [18]. However, these methods have several disadvantages such as expensive equipment, misquantification, complex procedure, and long turnaround time. In contrast, biosensors have attracted much attention from researchers in recent years and possess advantages such as portability, low cost, high sensitivity, and operation convenience [19], showing excellent application potential in on-site and fast-responding tests [20].

The core of a biosensor is a target recognition and sensing mechanism. The recognition elements include aptamers, antibodies, enzymes, etc. [21,22]. An aptamer is a short RNA or single-stranded DNA oligonucleotide with a specific three-dimensional structure for binding targets [23,24]. Compared with other probes, they have small and flexible structures with lower cost, better stability, better modifiability, and equivalent or higher selectivity and affinity for targets [25,26]. For sensing mechanisms, colorimetry, fluorescence, electrochemistry, and photonic crystal-based methods were reported for KanR detection in milk, with the limits of detection (LODs) from pM to tens of nM, and the turnaround time of tens of minutes [27,28,29,30,31,32]. The cause for the long turnaround time of these biosensors was the absence of effective means for target enrichment. In the last decade, microfluidic techniques have become versatile strategies for nanoparticle driving and separation, providing a good solution for target enrichment for biosensors, and shortening the turnaround time. However, most reported microfluidic methods depend on microchannels and pumps, making the system complex. Therefore, simple microfluidics with electrical excitation is becoming a promising technique coupled to electrical biosensors, particularly for on-site and real-time detection of KanR.

In this work, a microfluidic capacitive aptasensor is developed for KanR detection in milk. By using an aptamer as the probe, a PCB interdigitated electrode (IDE)-based sensor is constructed, employing a pF-level-resolution AC interfacial capacitance as the indicator. Meanwhile, the microfluidic AC electrothermal (ACET) effect is stimulated by the same AC signal for driving and enriching the target towards the electrode. Based on these sensing mechanisms and microfluidic effects, high sensitivity and short overall detection time are achieved. The turnaround time is 60 s, the linearity is over a wide range of 0.1 fM~1 pM, and the LOD is as low as 16.56 aM. The key performance of this aptasensor is compared with typical reported assays for KanR in milk, as presented in Table 1, showing a good competitiveness for field and real-time detection of milk KanR.

## 2. Materials and Methods

### 2.1. Reagents

A 5′-amino-modified aptamer for KanR was synthesized by Sangon Biotech Co., Ltd., Shanghai, China, according to previous studies, with the sequence of 5′-TGG GGG TTG AGG CTA AGC CGA-3′ [33]. It was diluted to 2.5 μM with phosphate buffer saline (0.05× PBS), purchased from Biosharp Co., Ltd., Shanghai, China. 6-Mercapto-1-hexanol (6-MCH) was purchased from Aladdin Biotechnology Co., Ltd., Shanghai, China, and was diluted to 1 mM with 0.05× PBS. KanR was purchased from Sangon Biotech Co., Ltd., Shanghai, China. Other antibiotics of penicillin G (PG), tobramycin (TOB), chloramphenicol (CL), streptomycin (STR), and gentamicin sulfate (GEN), were all purchased from Bioshun Biotech Co., Ltd., Shanghai, China.

### 2.2. Sample Treatment

For sensor calibration, all the above antibiotics, including KanR, were diluted in 0.1× PBS to obtain six concentrations from 0.1 fM to 10 pM, each with a 10-fold increase. The interfering proteins and antibiotics, including peptidoglycan (PG), tobramycin (TOB), chloramphenicol (CL), streptomycin (STR), and gentamicin (GEN), were prepared with the same process. The qualified skimmed milk was purchased from a local supermarket. To eliminate the interference of proteins and other impurities, the supernatant was collected after a milk sample was centrifuged at 12,000 rpm for 10 min, and this operation was repeated twice. The supernatant was then filtered through a sterile microporous membrane with an aperture of 0.22 μm. Subsequently, the obtained milk samples in triplicate were spiked with three KanR concentrations. Then, these samples were 1:10^6^ diluted with 0.1× PBS for the final test. The recovery rate can be calculated as follows: Recovery(%) = (Detected concentration − Original concentration)/Spiked concentration × 100%.

### 2.3. Aptasensor Preparation

**Figure 1:**
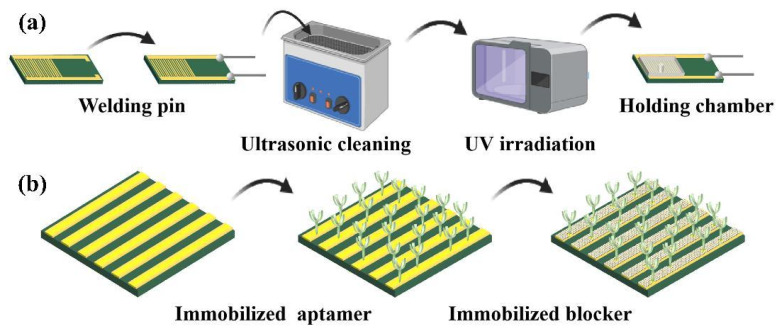
Process of sensor preparation. (**a**) Pretreatment before the sensor functionalization. (**b**) Sensor functionalization including aptamer and blocker immobilization.

As illustrated in Figure 1a, the basic structure of the sensor is a gold IDE fabricated by standard PCB process, with an external dimension of 3 cm × 1.5 cm, electrode width of 0.2 mm, and electrode gap of 0.15 mm. For its fabrication, the FR-4 (glass fiber-reinforced epoxy resin) is adopted as a substrate, with a covered copper layer of 35 μm (2 oz). After a thorough cleaning and removal of the surface oxide, graphical photoresist of about 30 μm is deposited and exposed. Then, the copper layer is etched to form the IDE pattern. Subsequently, the gold film is electroplated on the copper electrodes with the aid of a nickel bonding layer. The thicknesses of the nickel layer and gold layer are 3 μm and 1 μm, respectively. This IDE is fabricated by Shenzhen Jialichuang Technology Co., Ltd., Shenzhen, China. After soldering a pair of pins, the IDE is ultrasonically cleaned in acetone, isopropanol, and DI water for 3 min to completely remove the surface contaminants. Then, it is treated with ultraviolet light for 40 min to increase hydrophilicity. Finally, a silicone patch with a pore size of 4 mm is affixed to create a 20 μL chamber for holding the sample solution.

Figure 1b illustrates the sensor functionalization process. First, 20 μL of 2.5 μM aptamer for KanR is dropped into the chamber and incubated in a thermostatic humidor at 22 °C for 18 h, which facilitates the covalent bonding of the amino group at the 5′ end of the aptamer to the gold surface. After washing to remove the free aptamer, 20 μL of 1 mM 6-MCH is dropped and incubated at 18 °C for 2.5 h, blocking the blank sites at the IDE surface not occupied by the aptamer. After that, the IDE is cleaned again with DI water and stored at 4 °C for future use.

### 2.4. Enrichment Mechanism by ACET Effect

The ACET effect refers to a microfluidic phenomenon induced by a non-uniform electric field applied across the microelectrodes in a solution [34]. The current passing through the solution generates Joule heat, causing a local temperature increase, which affects the electrical conductivity and dielectric constant of the solution, resulting in their non-uniform distribution in space. Then, the coupling of the electric field and the temperature gradient generates the electrothermal volume force [35]. Therefore, subsequent micro-vortices appear, which can drive the embedded small particles and continuously transport them towards the electrode surface. Obviously, higher conductivity leads to more powerful vortices. In this work, the background of 0.1× PBS or the highly diluted milk by PBS has a high conductivity of about 0.184 S/m. Therefore, ACET flows are prominent for KanR molecule enrichment. The ACET force in the fluid is approximately expressed as

(1) $$F_{ACET}=0.011\frac{\varepsilon_{m}\sigma_{m}}{k}\pi rE_{rms}^{4}$$

where *ε_m_* (F/m), *σ_m_* (S/m) and *k* (W·m^−1^·K^−1^) are the permittivity, conductivity, and thermal conductivity of the fluid, respectively. *r* (m) is half of the electrode gap, and *E_rms_* (V/m) is the root mean square value of the electric field [36]. According to this equation *E_rms_* positively related to the applied voltage, playing a dominant role in enhancing the ACET effect.

### 2.5. Interfacial Capacitive Sensing

**Figure 2:**
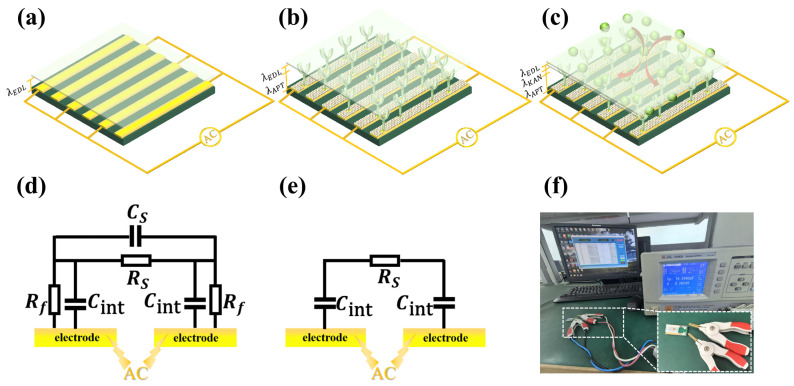
(**a**–**c**) Capacitive sensing mechanism of the aptasensor. (**d**,**e**) Equivalent circuit of the electrode–solution system. (**f**) Picture of the detection system.

When an electrode is immersed in an electrolyte, net charges are induced on its surface. Meanwhile, a counter-ion layer in the solution will be generated above the surface. These two layers form an electrical double layer (EDL), which acts as an interfacial capacitor with the intervening dielectric medium [35], as shown in Figure 2a. The interface capacitance C_int_ (F) is expressed as

(2) $$C_{\mathrm{int}}=\frac{\varepsilon_{EDL}S_{\mathrm{int}}}{\lambda_{EDL}}$$

where *S*_int_ (m^2^) is the surface area of interface capacitance, *ε_EDL_* (F/m) is the solution permittivity, and *λ_EDL_* (m) is the thickness of EDL. When the aptamer is immobilized on the electrode surface, the thickness of the dielectric layer containing the EDL becomes thicker. Therefore, the interfacial capacitance will change, as shown in Figure 2b. This change can reflect the binding of KanR to the aptamer, and further indicates the KanR concentration in the solution. The changed capacitance C_int-apt_ (F) can be expressed as

(3) $$C_{\mathrm{int-}\mathrm{apt}}=\frac{S_{\mathrm{int-}\mathrm{apt}}}{\frac{\lambda_{EDL}}{\varepsilon_{EDL}}+\frac{\lambda_{APT}}{\varepsilon_{APT}}}$$

where S_int-apt_ (m^2^) is the surface area after aptamer immobilization, *ε_APT_* (F/m) is the permittivity of the aptamer, and *λ_APT_* (m) is the thickness of the aptamer layer. As illustrated in Figure 2c, the ACET vortices will drive the KanR molecules to the IDE surface and bind with the aptamer. Then, the thickness of capacitor Cint-combine is superposed by the KanR, aptamer, and EDL, expressed as

(4) $$C_{\mathrm{int-}\mathrm{combine}}=\frac{S_{\mathrm{int}-\mathrm{combine}}}{\frac{\lambda_{EDL}}{\varepsilon_{EDL}}+\frac{\lambda_{APT}}{\varepsilon_{APT}}+\frac{\lambda_{KAN}}{\varepsilon_{KAN}}}$$

where *ε_KAN_* (F/m) is the permittivity of KanR, *λ_KAN_* (m) is the thickness of the KanR layer, and *S*_int-combine_ (m^2^) is the surface area after target binding. Assuming the same surface area during the detection process, the normalized change rate of the capacitance during the detection is given as

(5) $$\frac{\Delta C}{C_{\mathrm{int-}\mathrm{apt}}}=\frac{C_{\mathrm{int-}\mathrm{combine}}-C_{\mathrm{int-}\mathrm{apt}}}{C_{\mathrm{int-}\mathrm{apt}}}=-\frac{\lambda_{KAN}}{\frac{\varepsilon_{KAN}}{\varepsilon_{EDL}}\lambda_{EDL}+\frac{\varepsilon_{KAN}}{\varepsilon_{APT}}\lambda_{APT}+\lambda_{KAN}}$$

where ΔC is acquired in a duration of 1 min. According to Equation (5), $\frac{\Delta C}{C_{\mathrm{int}-\mathrm{apt}}}$, i.e., the dC/dt (%/min) in the later section, has no connection with the initial capacitance, avoiding the test error caused by the inconsistent initial state of different sensor surfaces.

The electrode–electrolyte system can be approximately described by the equivalent circuit network in Figure 2d, where *C*_int_ and *R_f_* represent the interfacial capacitance and charge transfer resistance at the electrode–fluid interface, respectively, and *R_s_* and *C_s_* are the bulk resistance and capacitance of the solution, respectively. When the AC frequency is lower than 100 kHz, *C*_int_ and *R_s_* play a dominant role in this network [37]. Therefore, the equivalent circuit can be simplified as a series connection of two *C*_int_ and one *R_s_*, as shown in Figure 2e. The observable variables of *C*_int_ just reflect the process of KanR binding to the aptamer. The detection schematic is illustrated in Figure 2f, with an impedance analyzer (Tonghui TH2828, Changzhou, China) for applying the AC excitation and testing the capacitance.

## 3. Results and Discussion

### 3.1. Sensor Characterization

**Figure 3:**
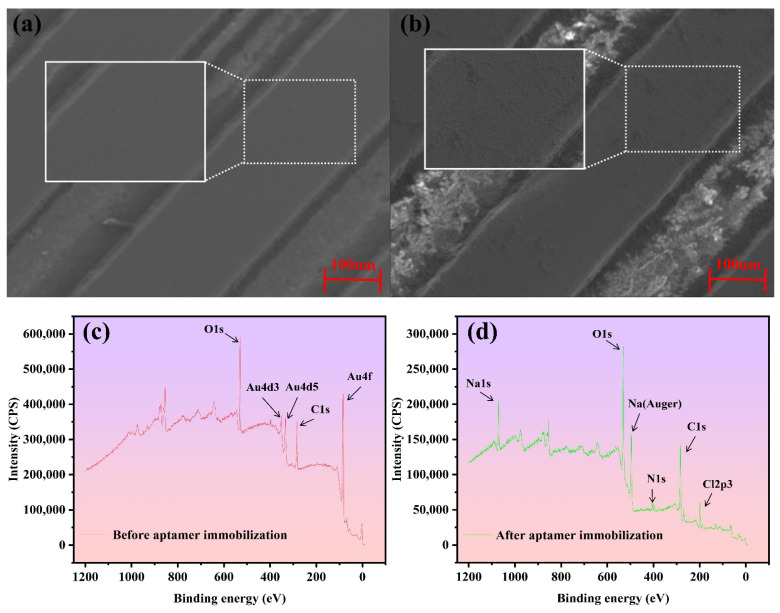
(**a**,**b**) SEM images and (**c**,**d**) XPS results of the sensor before and after functionalization, where the zoom-in views are in the white solid line frames corresponding to the areas within the white solid line frames.

The sensor functionalization is examined by observing the IDE surface with a scanning electron microscope (SEM) as shown in Figure 3a,b, where a layer of homogenous and slightly rough material is clearly found after the aptamer and blocker coating, indicating successful immobilization. As the electrode gaps do not play a role in the formation of capacitance, the deposition on the gaps has no effect on the sensing. In addition, X-ray photoelectron spectroscopy (XPS) is utilized for characteristic element identification before and after aptamer immobilization, as shown in Figure 3c,d. The element of Au from the IDE surface is dominant before aptamer coating, while after aptamer immobilization, it can hardly be found with the appearance of N, Na, and Cl, which are from the aptamer and PBS. This result indicates a good modification of the IDE by the aptamer.

**Figure 4:**
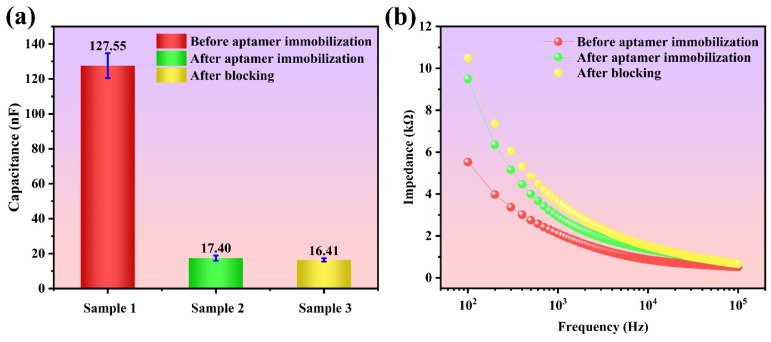
Change of (**a**) capacitance and (**b**) impedance spectroscopy before and after aptamer immobilization and after blocking.

Using the electrical method, the sensor functionalization can further be examined. The capacitance and the impedance spectroscopy are measured before and after aptamer immobilization and after blocking, as shown in Figure 4a and Figure 4b, respectively. The capacitance significantly decreases after aptamer immobilization. Because the 6-MCH molecules are much smaller than aptamers, they do not generate an apparent additional layer for the interfacial capacitance. Therefore, the capacitance change from the blockers is slight. As for the impedance, it increases after these functionalizations due to the smaller conductivity of these overlays. Analogically, the change from aptamers is more significant than that from blockers. In conclusion, these electrical results demonstrate good functionalization of the IDE.

### 3.2. Optimization of Excitation Signal

**Figure 5:**
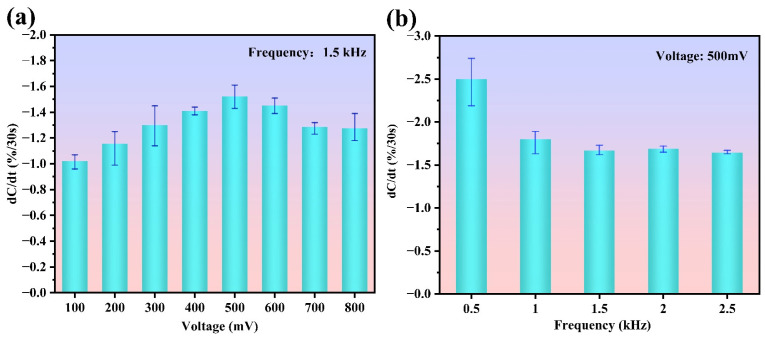
Optimization of (**a**) voltage and (**b**) frequency of the excitation signal for test.

As indicated in Equation (2), the ACET force has a strong positive correlation with the electric field. Therefore, a high applied voltage will enhance the target enrichment. However, too high a voltage may increase the possibility of premature adsorption saturation and undesired nonspecific adsorption. Under a fixed AC frequency of 1.5 kHz, a KanR standard solution of 1 fM is detected at different voltages (from 100 mV to 800 mV with a step of 100 mV). Each test is repeated three times to obtain the average response using three new sensors. In the results, as shown in Figure 5a, the response monotonically increases with the voltage from 100 mV to 500 mV. However, when the voltage is above 500 mV, the response begins to decrease due to the adsorption saturation. Thus, 500 mV is adopted as the optimized voltage.

To further determine an appropriate frequency, different frequencies (from 0.5 kHz to 2.5 kHz with a step of 0.5 kHz) are set for the KanR test, with an AC voltage of 500 mV. As shown in Figure 5b, the average response from the sensor decreases as the frequency increases. Although the sensor produces a larger response at the frequencies of 0.5 kHz and 1 kHz, the error bar is significant, indicating poor stability. Therefore, 1.5 kHz is confirmed as the optimized frequency for future detection.

### 3.3. Dose Response and Sensor Calibration

**Figure 6:**
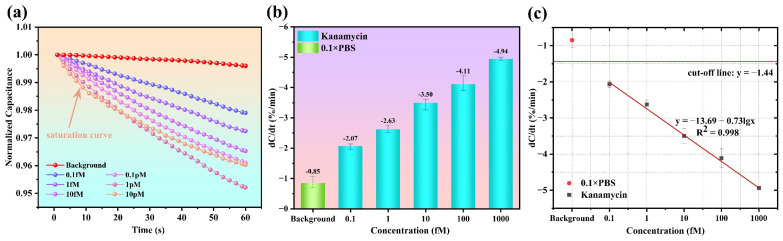
(**a**) Sensor calibration. (**b**) Fit curve of the dose response. (**c**) Normalized transient capacitance curves from KanR of different concentrations.

Triplicate detection is performed with the 0.1× PBS background and KanR standard solutions at six concentrations, as specified. The typical normalized transient capacitance curves in 60 s are as shown in Figure 6a, exhibiting a clear distinction between different concentrations. The absolute slope of the transient curves gradually increases as the KanR concentration rises. However, when the concentration reaches 10 pM, an upswept curve is observed, indicating a saturated status at the IDE. Therefore, the linear dynamic range of this aptasensor is determined as (0.1 fM~1 pM). Additionally, the blank control of 0.1× PBS produces an almost unchanging curve.

Based on the transient curves, a series of fitted slopes can be obtained, which are just the previously defined response of dC/dt (%/min) indicating the KanR concentrations. As shown in Figure 6b, the dose responses with their standard deviations (STDEVs) are −2.0 ± 0.09%/min, −2.63 ± 0.12%/min, −3.50 ± 0.20%/min, −4.11 ± 0.25%/min, −4.94 ± 0.05%/min, and −4.30 ± 0.30%/min, corresponding to the concentrations of 0.1 fM, 1 fM, 10 fM, 0.1 pM and 1 pM, respectively.

To provide a reference for the future detection of milk samples with unknown concentrations of KanR, calibration for this aptasensor is necessary. Using the obtained responses, a linear fitting is performed using the least squares method. As shown in Figure 6c, the calibration equation is y(%/min) = −13.69 − 0.73lgx(mol/L), with the correlation coefficient of R^2^ = 0.998. After a cut-off line is defined as a response of that from 0.1× PBS minus three times the STDEV (towards the negative *Y*-axis), there is an intersection point of the calibration line and the cut-off line, with the response value known as the limit of detection (LOD), being a very low concentration of 16.56 aM.

### 3.4. Sensor Specificity

**Figure 7:**
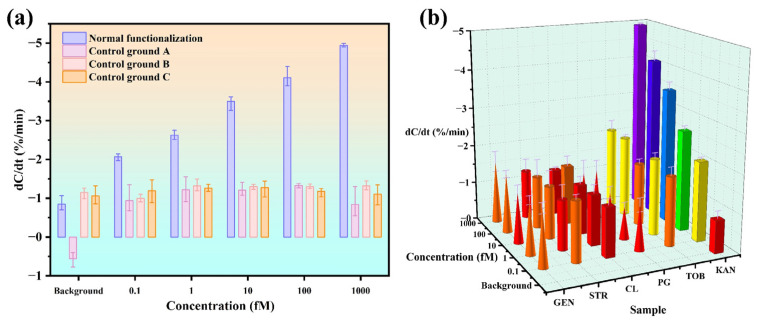
Specificity of the sensor. (**a**) Response of functionalized and dummy sensors from KanR. (**b**) Response to KanR against the interferences.

To verify the sensor specificity, three groups of dummy sensors are prepared as controls. Group A is a set of sensors modified without a KanR aptamer but with a blocker. Groups B and C are sensors with the probes replaced by a Micro RNA-221 aptamer and a cov-N58 aptamer, respectively, ordered from Sangon Biotech Co., Ltd., Shanghai, China. Using the above three types of sensors and the sensors for KanR, standard samples of KanR in 0.1× PBS are detected. As shown in Figure 7a, the sensors for KanR show excellent dose–response, while all the dummy sensors produce similar responses to the background without any significant changes. This indicates that the aptamer for KanR is qualified and the aptasensors are successfully functionalized.

Next, common interfering proteins and antibiotics of PG, TOB, CL, STR, and GEN are tested using the aptasensors to evaluate the specificity, together with the KanR from 0.1 fM to 1 pM. It can be seen in Figure 7b that even for TOB, STR, and GEN, as the same aminoglycoside antibiotics as KanR, the responses at the highest concentration are still much lower than that from KanR at the low concentration of 0.1 fM. Among all the interferences, TOB shows the maximum response of −2.11%/min. Using the calibration equation, the same response from KanR corresponds to a concentration of 0.129 fM. Therefore, the selectivity of this sensor can be calculated as 7752:1 (1 pM:0.129 fM). According to these analyses, the aptasensor demonstrates excellent selectivity for KanR.

### 3.5. Repeatability and Long-Term Stability of the Sensor

**Figure 8:**
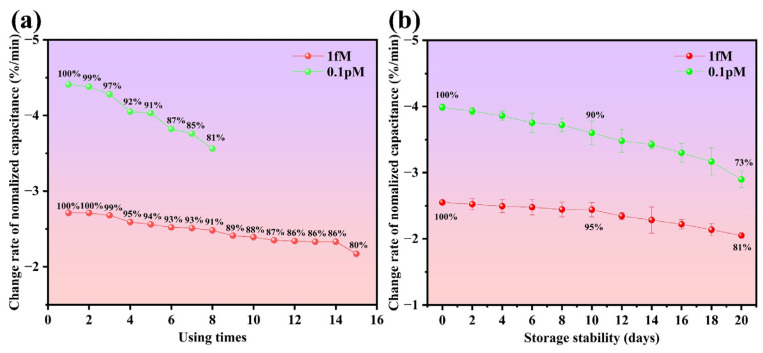
Repeatability and long-term stability of the sensor. (**a**) Response degradation of the sensor after being reused multiple times. (**b**) Response degradation of the sensors after being stored at 4 °C for different days.

Repeatability refers to the capability of a sensor to maintain consistency in repeated measurements. Although the aptasensor in this work is designed for single use, good repeatability can still reduce the cost. Due to the short detection duration, the aptamer sites might not be occupied by the targets. After setting 90% of the desired responses as the threshold, the sensors are retested. As shown in Figure 8a, when a sensor is used 5 times to detect a 0.1 pM standard sample, its response drops to 91%. Meanwhile, when detecting a 1 fM standard sample, the response remains about 91% until 8 repeated detections. These data indicate that the reusability for higher-concentration samples is worse. As an estimate, this sensor can be reused 5 times if the KanR concentration is not ultrahigh.

Long-term stability refers to the shelf life of the sensor under certain storage conditions. In this work, aptasensors of different batches are stored in a refrigerator at 4 °C, and the threshold response is also set as 90% of the desired response. On the 10th day, as shown in Figure 8b, the responses from KanR with the concentrations of 0.1 pM and 1 fM drop to 90.33% and 95.15%, respectively, which is mainly caused by the inactivation of the aptamer. Therefore, this aptasensor can maintain good performance when stored at 4 °C for about 10 days.

### 3.6. Application with Milk Samples

Because foods with excessive levels of antibiotics are hard to obtain, qualified skimmed milk is tested after being spiked with KanR to investigate the applicability as well as the performance of this sensor for practical samples. All the following tests are performed after dilutions with the procedure described previously. First, the milk without spiking is detected to determine the original KanR concentration of 3.13 fM. Subsequently, triplicate milks (990 mL each) are spiked with 10 mL KanR at the concentrations of 153 fM, 303 fM, and 603 fM, respectively. The three theoretical concentrations are then obtained as 4.63 fM, 6.13 fM, and 9.13 fM, respectively. Using the aptasensors, the detected KanR concentrations of the spiked samples are 4.58 fM, 6.65 fM, and 8.37 fM, respectively. According to the theoretical and detected values, the recovery of three spiked samples is calculated as shown in Table 2, from 86.90% to 116.17%, with a relative standard deviation (RSD) from 2.88% to 3.60%, demonstrating satisfactory accuracy and stability of this sensor for practical application.

## 4. Conclusions

Rapid and sensitive detection of antibiotics in complex matrices is of great significance to food safety. At present, most sensors or assays are still facing the challenges of real-time responding and particularly when the antibiotic concentration is low, requiring necessary pre-enrichment or extraction steps. Taking KanR as a typical antibiotic commonly found in dairy products, an aptasensor is developed integrating an ultrasensitive interfacial capacitive sensing mechanism with powerful microfluidic ACET enrichment. Thus, the sub-femtomolar LOD and 60 s turnaround time are realized simultaneously, independent of extra pretreatment except simple dilution and filtration. Owing to the ultralow LOD, a large amount of interferences can be removed in the matrices via dilution, while the concentrations for the test are still within the sensor’s dynamic range, which covers five orders of magnitude. With high specificity, low cost, and simple operation, this aptasensor is successfully applied for KanR detection in milk, demonstrating good feasibility for real sample detection. In conclusion, this aptasensor provides a competitive solution for real-time, highly sensitive, and low-cost KanR detection in food.

## Figures and Tables

**Table 1:** Comparison of recent assays for KanR detection in milk.

Methods	Turnaround Time	Linear Range	LOD	Sample	Reference
Colorimetry	–	0.02–0.1 μM	8 nM	Milk	[27]
Colorimetry	55 min	25–800 nM	20.58 nM	Tap water, Milk	[28]
Fluorescence	–	1–400 nM	0.60 nM	Milk	[29]
Fluorescence	–	100–1100 nM	22.6 nM	Milk	[30]
Electrochemistry	120 min	50 nM–1 μM	6.044 nM	Milk	[31]
Photonic crystal	–	10.3 pM–10.3 μM	2.3 pM	Milk	[32]
Capacitance	1 min	0.1 fM–1 pM	16.56 aM	Milk	This work

**Table 2:** Detection of KanR in milk samples.

Sample	Spiked Concentration (fM)	Theoretical Concentration (fM)	Detected Concentration (fM)	Recovery (%)	RSD (%, *n* = 3)
Skimmed milk	1.53	4.63	4.58	94.77	2.88
	3.03	6.13	6.65	116.17	3.60
	6.03	9.13	8.37	86.90	2.94

## Data Availability

Data supporting the findings of this study are available from the corresponding author H. Qi upon request.
